# Camel Milk and Its Bioactive Components in Diseases Associated with Aging: A Narrative Review

**DOI:** 10.3390/nu18172935

**Published:** 2026-09-07

**Authors:** Mengwei Cheng, Tianyan Liu, Xixian Wang, Situ Xue

**Affiliations:** 1 Institute of Medicinal Biotechnology, Chinese Academy of Medical Sciences and Peking Union Medical College, Beijing 100050, China; 2 Plentiful Pasture, Yili Fengcao Muchang Dairy Co., Ltd., Ili 835221, China

**Keywords:** camel milk, nutrition, bioactive components, aging, anti-inflammation

## Abstract

Camel milk has long been used as a traditional health-promoting food, and growing biomedical evidence now supports its modulatory effects across multiple age-related conditions. This narrative review evaluates current experimental and clinical findings on the effects of camel milk across major categories of age-related disease, spanning metabolic dysregulation, cardiovascular pathology, neurological dysfunction, and immune-inflammatory conditions. Across these conditions, camel milk has been reported to modulate oxidative stress, chronic inflammation, and metabolic homeostasis through shared molecular pathways, including NF-κB, MAPK, PI3K/AKT, and PPAR-α/γ signaling. Mapping these effects against the twelve hallmarks of aging reveals particular engagement with chronic inflammation, deregulated nutrient sensing, and dysbiosis. Collectively, the available evidence supports a biologically plausible role for camel milk in modulating key pathological processes associated with age-related disease. However, the clinical evidence remains limited across most disease areas, and future research should prioritize standardized clinical trials and systematic mechanistic validation, with particular attention paid to the bioavailability and dose–response characterization of key bioactive components. This review provides an integrative synthesis of camel milk research within the hallmarks of the aging framework, offering a cross-disease mechanistic perspective that extends beyond single-disease or compositional approaches.

## 1. Introduction

Camels are mainly distributed in arid and semi-arid regions such as North Africa, the Middle East and Central Asia, and are one of the important livestock resources in these regions [1]. At present, the global camel population continues to grow, and its adaptability to extreme ecological environments makes it irreplaceable in local agricultural and pastoral systems [1,2]. Unlike dairy cattle, which have been intensively selected for sustained year-round lactation, the initiation and maintenance of lactation in camels depend closely on reproductive status and environmental conditions [2,3]. For a long time, camel milk has been an important nutritional resource in camel-raising areas, providing abundant energy, high-quality protein and essential micronutrients [4]. In addition to basic nutritional value, camel milk also contains a variety of bioactive components, including casein components, whey protein, lactoferrin (LF), immunoglobulins and vitamins [5]. The potential biological activity exhibited by these components has made them a focus of research in the fields of medicine and nutrition in recent years. It should be noted, however, that camel milk is not compositionally constant. Breed, feeding regimen, geographical origin, lactation stage, processing and fermentation, and storage conditions can all affect its composition [2,3].

Camel milk has been widely utilized for health-related purposes across diverse cultures for centuries. In classical Chinese medical texts and in the medical literature of the Uyghur and Kazakh peoples of Xinjiang, camel milk is recorded as a resource of both dietary and medicinal value [6]. In the Middle East, North Africa and Central Asia, it has likewise long been used as a traditional remedy against infectious and skin diseases and as a tonic to enhance physical strength and resistance [7,8,9]. The long-term, widespread and relatively consistent ethnic medical practices in different regions suggest that camel milk merits further study in regulating physical strength and immune function and maintaining health related to chronic diseases, and provide an important empirical basis for its application in the field of healthy aging.

In recent decades, modern biomedical research has increasingly focused on the biological and therapeutic properties of camel milk. In vitro experiments, animal models, and a growing body of clinical and observational studies have shown that camel milk may play beneficial roles in metabolic regulation, inflammatory responses, oxidative stress, immune function, and organ protection [10,11]. It has been investigated in age-associated conditions, including type 2 diabetes, cardiovascular disease, inflammatory diseases, osteoporosis, and neurodegenerative diseases [12,13,14]. The unique composition of camel milk may be an important material basis for its above-mentioned health effects. It is rich in a variety of bioactive proteins, including LF, lysozyme, immunoglobulins, and insulin-like proteins, which have antibacterial, anti-inflammatory, and immunomodulatory functions. At the same time, camel milk contains high levels of vitamin C and a variety of antioxidants, which help alleviate oxidative stress. In addition, its fatty acid composition and mineral content are also closely related to metabolic regulation and bone health [15,16,17], suggesting that camel milk may intervene in aging-related biological processes through these active ingredients, providing important evidence for nutritional strategies for healthy aging.

In this review we distinguish the following concepts: biological aging refers to the progressive accumulation of molecular and cellular damage over time; age-associated disease refers to diseases whose prevalence increases with age; and healthy aging refers to the maintenance of functional ability with advancing age. All of the conditions covered here are age-associated diseases, and in the great majority of the studies cited, aging itself was not an experimental variable.

Based on the above background, this narrative review summarizes the effects of camel milk and its isolated bioactive constituents on aging-related metabolic, cardiovascular, neurodegenerative, and immune-inflammatory diseases. For each category, evidence is presented from human studies to animal and cellular models, and further to the underlying molecular mechanisms. Through this structured approach, the review aims to clarify the therapeutic potential of camel milk and its regulatory roles in aging-associated pathways, while also identifying current limitations and outlining future research directions. Whole camel milk has been used in most human and animal studies, whereas much of the mechanistic evidence derives from isolated constituents such as lactoferrin, hydrolysate-derived peptides, and extracellular vesicles; these are not equivalent in dose or route of exposure, and the distinction is maintained throughout the following sections.

### Literature Search Strategy and Scope

We conducted a structured literature search in PubMed, Web of Science Core Collection, Scopus, and Google Scholar databases, covering the period from the databases’ inception to August 2026. Search terms combined terms related to camel milk and its bioactive components (“camel milk”, “camel lactoferrin”, “camel milk extracellular vesicles”, and “camel milk exosomes”) and terms related to aging and the disease areas covered in this review, including “aging”, “age-related”, “healthy aging”, “diabetes”, “metabolic diseases”, “cardiovascular diseases”, “atherosclerosis”, and “inflammation”. We also screened relevant reviews and reference lists of articles meeting the inclusion criteria to identify other research. We referenced the literature on studies and reviews of whole camel milk, processed or fermented camel milk, or well-defined camel milk derivatives, categorized by human studies, animal studies, and in vitro studies.

Research related to camel milk also covers areas such as cancer, infectious diseases, and pediatric or neurodevelopmental disorders. Because this review focuses on chronic diseases and functional impairments with increasing prevalence or clinical burden with age, these areas were not included. As this is a narrative review, study selection and evidence synthesis were conducted using qualitative methods; no formal risk assessment or meta-analysis was performed.

## 2. Metabolic Diseases

Metabolic and endocrine diseases are chronic conditions characterized by dysregulation of energy metabolism and endocrine function, typically manifested by abnormalities in glucose and lipid metabolism and impaired insulin signaling. Their prevalence increases markedly with age. For example, the prevalence of type 2 diabetes in individuals aged 60 years and older can approach or exceed 20%, while the detection rate of metabolic syndrome in those aged 65 years and older often reaches 40% or higher across multiple populations [18,19]. Pathophysiologically, these disorders are primarily driven by insulin resistance, chronic low-grade inflammation, oxidative stress, and disrupted energy homeostasis [5,20,21]. Insulin-like proteins, lactoferrin, immunoglobulins, bioactive peptides, and various antioxidants in camel milk have been shown to be closely related to the development and progression of metabolic and endocrine system diseases. Previous studies have indicated that these components can participate in the regulation of glucose and lipid metabolism, the alleviation of oxidative stress, and the inhibition of inflammatory signaling pathways at multiple levels, thus exhibiting multi-target and comprehensive regulatory potential in metabolic-related pathological processes [5].

### 2.1. Diabetes

At the clinical level, multiple randomized controlled trials have demonstrated that long-term camel milk supplementation, as an adjunct to conventional treatment, may contribute to improved glycemic management in diabetic patients. Two randomized controlled trials have examined camel milk as an adjunct to conventional therapy in patients with type 1 diabetes mellitus (T1DM) over extended periods. A 16-week randomized clinical study enrolling 54 T1DM patients reported significant reductions in fasting blood glucose (98.9 ± 16.2 vs. 227.2 ± 17.7 mg/dL), glycated hemoglobin (HbA1c) levels (7.16 ± 1.84% vs. 9.59 ± 2.05%), and daily insulin requirements following daily consumption of 500 mL of camel milk compared with standard management alone. Serum C-peptide was also measured in that study and did not differ significantly between groups, indicating that these improvements are unlikely to reflect increased endogenous insulin secretion; the underlying mechanism remains to be defined [22]. A subsequent 2-year randomized clinical trial reported findings in the same direction over a longer observation period: 24 patients with T1DM were allocated to usual care (*n* = 12) or usual care plus 500 mL/day of camel milk (*n* = 12). In the camel milk group, mean blood glucose fell from 118.58 ± 19 to 93.16 ± 17.06 mg/dL, HbA1c from 7.81 ± 1.39% to 5.44 ± 0.81%, and insulin dose from 32.50 ± 9.99 to 17.50 ± 12.09 U/day (*p* < 0.05) [23].

A number of randomized controlled trials have since been conducted in patients with Type 2 diabetes mellitus (T2DM) using fresh milk, milk powder and probiotic-fermented preparations, with intervention periods ranging from four weeks to ten months. In a trial of 60 patients with T2DM already receiving oral antidiabetic medication, addition of 500 mL/day of raw camel milk for three months was accompanied by a fall in HbA1c from 9.44 ± 0.16% to 6.61 ± 0.14% (a reduction of approximately 30%, *p* < 0.05), together with significant decreases in total cholesterol and triglycerides; urea and creatinine did not differ between groups [24]. A double-blind, placebo-controlled clinical trial conducted in type 2 diabetic patients reported that 4-week supplementation with camel milk powder (10 g twice daily; *n* = 14, with cow milk powder as placebo, *n* = 13) led to decreases in fasting blood glucose, postprandial blood glucose, and total cholesterol, alongside favorable changes in adipokine profiles, together with within-group increases in serum osteocrin, amylin and glucagon-like peptide-1 (GLP-1), and enrichment of specific gut taxa relative to the cow milk group [25]. Several further trials using 500 mL/day of fresh camel milk over periods of six months or less have reported reductions in triglycerides, with differences in total cholesterol and other lipid fractions not reaching significance [26], whereas a ten-month study reported significant decreases in both total cholesterol and triglycerides [26]. Taken together, the human evidence in T1DM centres on reductions in insulin requirement and HbA1c, whereas in T2DM it centres on glycaemic and lipid parameters, with effect sizes that appear to vary with intervention duration and product form.

In animal models, camel milk intervention has also been observed to improve hyperglycemia and increase insulin sensitivity. Early studies suggested that this hypoglycemic effect was largely attributed to the “insulin-like components” contained in camel milk [27]. In this regard, the hypoglycemic activity of camel milk has been partly attributed to its insulin-like proteins or bioactive peptides, which are presumed to survive gastrointestinal digestion to some extent and interact with insulin-related signaling pathways. However, the exact identity, concentration, and oral bioavailability of these components remain incompletely defined [27]. In receptor-level studies conducted in vitro, camel milk has been reported to enhance the interaction between the insulin receptor (IR) and growth factor receptor-bound protein 2 (Grb2), rather than IRS-1, thereby preferentially activating the extracellular signal-regulated kinase 1/2 (ERK1/2) signaling pathway. This leads to increased ERK phosphorylation and amplification of insulin-induced responses. Meanwhile, it also synergizes with the PI3K/AKT pathway to upregulate glucose transporter type 4 (GLUT4) expression and promote glucose uptake [27]. Notably, these signaling effects appear to be dose- and matrix-dependent, as studies comparing raw and heat-treated camel milk have reported differential impacts on IR activation, highlighting the importance of processing conditions in preserving bioactive protein integrity [28]. In the pathological process of diabetes, oxidative stress and chronic inflammation are not only the result of metabolic disorders, but also important driving factors that further aggravate insulin resistance and β-cell damage. Persistently elevated levels of ROS can damage the structure and function of pancreatic β-cells and activate multiple pro-inflammatory signaling pathways, thereby interfering with insulin signal transduction [29]. In streptozotocin-induced diabetic rats, camel milk protein hydrolysates lowered blood glucose and lipid levels and attenuated the accompanying oxidative stress [30]; in a rat model of diabetic nephropathy, camel milk and its exosomes were likewise reported to improve oxidative and inflammatory indices [31]. Findings such as these are consistent with the view that LF and other bioactive components in camel milk have significant antioxidant and immunomodulatory capabilities, which can reduce lipid peroxidation levels and regulate the expression of inflammatory factors [22,32,33]. In addition, the iron-chelating capacity of LF may theoretically contribute to reduced oxidative burden by limiting iron-catalyzed Fenton reactions and hydroxyl radical formation, although direct evidence in camel milk-treated diabetic tissues remains limited [34].

At the cellular level, LF isolated from camel milk can activate the IR and its downstream signaling pathways in an in vitro cell model, including enhancing the phosphorylation levels of the PI3K/AKT and ERK1/2 pathways, thereby promoting glucose uptake and energy metabolism-related processes [35]. That study examined lactoferrin from both camel and bovine milk, and both preparations induced IR, AKT and ERK1/2 phosphorylation in HepG2 and HEK293 cells, accompanied by increased glucose uptake. Because AKT signaling governs GLUT4 expression and translocation, the purified protein engages a branch of insulin signaling that whole camel milk did not [35]. In addition, camel milk-derived protein/peptide components have been reported to specifically enhance ERK1/2 phosphorylation at saturating insulin concentrations while improving β-cell structure and insulin secretion function [27]. The findings suggest that certain active protein components in camel milk may not simply replace insulin function, but rather participate in the fine regulation of glucose homeostasis by modulating IR and its downstream signaling networks. Camel milk-derived whey protein hydrolysates have been shown to inhibit dipeptidyl peptidase-4 (DPP-4) in vitro [36]. Serum GLP-1 was found to increase in the type 2 diabetes trial described above [25]; however, DPP-4 activity was not measured in that trial, and the relationship between the two observations remains speculative.

Besides directly affecting host metabolic pathways, camel milk may also indirectly influence glucose homeostasis by modulating gut microbiota composition and its metabolites. In a type 2 diabetic mouse model, camel milk peptides improved gut microbiota dysbiosis and altered fecal metabolic profiles. Correlation analysis suggested that its hypoglycemic effect may be related to changes in short-chain fatty acids and acidic metabolites caused by changes in gut microbiota [37]; studies have shown that camel milk intervention can improve the abundance of gut microbiota related to short-chain fatty acid production. Short-chain fatty acids (SCFAs), as important gut metabolites, can regulate insulin sensitivity and energy metabolism through mechanisms such as stimulating intestinal L cells to secrete glucagon-like peptide-1 (GLP-1), inhibiting hepatic gluconeogenesis, and promoting glucose uptake in peripheral tissues. Moreover, SCFAs can activate the AMP-activated protein kinase (AMPK) signaling pathway via G protein-coupled receptor 41/43 (GPR41/43) receptors, thereby further suppressing the expression of hepatic gluconeogenic enzymes such as phosphoenolpyruvate carboxykinase (PEPCK) and glucose-6-phosphatase (G6Pase) [37,38]. However, this inference has not yet been directly verified in camel milk research. In addition, camel milk intervention has been associated with enrichment of *Lactobacillus* and *Bifidobacterium* species and improved intestinal barrier function. In the T2DM rat model, the compound probiotics derived from fermented camel milk upregulated the expression of colonic tight junction protein and decreased serum lipopolysaccharide level. Given that toll-like receptor 4 (TLR4) is a key node connecting innate immunity and insulin resistance, the reduction in endotoxin exposure associated with improved barrier function may be one of the ways in which camel milk affects insulin sensitivity, but this pathway has not been directly tested in camel milk studies [39,40].

### 2.2. Nonalcoholic Fatty Liver Disease

Non-alcoholic fatty liver disease (NAFLD) is a metabolic liver disease characterized by abnormal accumulation of lipids in hepatocytes. In 2023, a multisociety consensus renamed the condition metabolic dysfunction-associated steatotic liver disease (MASLD) and adopted positive diagnostic criteria based on cardiometabolic risk factors; the original term is retained below when discussing earlier studies [41]. Its development is closely related to insulin resistance, lipid metabolism disorders, oxidative stress, gut microbial dysbiosis and chronic inflammatory response [42]. Deregulated nutrient sensing, mitochondrial dysfunction, impaired autophagy, and chronic low-grade inflammation are also recognized hallmarks of aging [12,43], providing a framework for interpreting the hepatoprotective effects of camel milk in the context of aging.

At the population level, the amount of direct clinical evidence on the intervention of camel milk in NAFLD is limited. Available trials have generally been conducted in high-risk populations such as individuals with metabolic syndrome rather than in patients with imaging- or histology-confirmed disease. A double-blind randomized crossover trial of 24 overweight or obese adolescents with metabolic syndrome showed that 250 mL/day of fermented camel milk for eight weeks, compared with an equal volume of diluted cow-milk yogurt, improved serum liver enzymes: aspartate aminotransferase (AST) decreased by 3.75 U/L (95% confidence interval [CI], −7.06 to −0.43), alanine aminotransferase (ALT) decreased by 2.54 U/L (95% CI, −3.33 to −2.24), and the AST/ALT ratio also decreased [44]. Glycemic, insulin-resistance, and inflammatory outcomes from the same trial were reported separately [45]. In addition, a review pointed out that incorporating camel milk into the daily diet may have beneficial effects on high-risk groups of liver disease (including NAFLD), mainly reflected in the improvement in liver function-related biochemical indicators, highlighting its potential as an adjunct nutritional intervention for NAFLD-associated hepatic dysfunction [46]. Moreover, fermentation may enhance these beneficial effects by generating bioactive peptides with angiotensin-converting enzyme (ACE)-inhibitory and antioxidant activities. These peptides are not present, or are present at much lower levels, in unfermented camel milk because enzymatic hydrolysis during fermentation releases peptide sequences from parent proteins such as casein and LF [44,47,48].

In animal models, the ameliorative effect of camel milk on NAFLD-related pathological changes has been observed more consistently. Korish et al. found in a rat model induced by a high-fat, high-cholesterol diet that continuous intake of camel milk for 8 weeks significantly reduced hepatic fat accumulation and inflammatory cell infiltration, accompanied by improvements in serum triglycerides and liver function indicators [49]. Mechanistically, camel milk, particularly through LF, upregulates antioxidant enzymes such as superoxide dismutase (SOD), catalase (CAT), and glutathione peroxidase (GPx); reduces lipid peroxidation as indicated by decreased malondialdehyde (MDA) levels; and inhibits the nuclear translocation of sterol regulatory element-binding protein 1c (SREBP-1c), thereby downregulating lipogenic enzyme expression. Meanwhile, it activates the PPARα/retinoid X receptor (RXR) heterodimer to promote carnitine palmitoyltransferase-1 (CPT1)-mediated fatty acid β-oxidation [48]. At the same time, this intervention also enhanced antioxidant enzyme activity and reduced lipid peroxidation product levels, suggesting that it can alleviate oxidative stress. In addition, related studies have reported that camel milk and camel milk hydrolysate (CMH) intervention can significantly reduce the degree of hepatic fat accumulation in high-fat diet-induced model animals, thus showing an anti-fatty liver effect [15,50]. At the histological level, hepatocyte fat vacuolar degeneration and inflammatory cell infiltration were significantly reduced in the liver tissue of the camel milk treatment group, further supporting its clear hepatoprotective effect in the NAFLD model [51]. Furthermore, camel milk-derived exosomes may remodel the gut microbiota, increasing the production of SCFAs such as butyrate, which in turn activate hepatic AMPK signaling via GPR43, Activated AMPK phosphorylates and inhibits acetyl-CoA carboxylase (ACC), thereby limiting de novo lipogenesis, and suppresses mTORC1 signaling, a change generally associated with de-repression of autophagy and autophagy-mediated (lipophagic) lipid-droplet clearance. It should be noted that the mTORC1 autophagy step reflects the general biological characteristics of AMPK, and there is no mechanism that can be directly verified by camel milk yet [32,52].

The molecular-level study suggests that the intervention effect of camel milk on NAFLD may mainly manifest in regulating the expression of lipid metabolism-related transcription factors and key enzymes, inhibiting hepatic lipogenesis and promoting fatty acid β-oxidation, thereby correcting the imbalance of lipid metabolism in the liver [48]. In addition, the various antioxidant components contained in camel milk can significantly reduce lipid peroxidation levels and alleviate the damage to hepatocyte structure and function caused by oxidative stress; among them, vitamin C in camel milk can act as an electron donor to directly scavenge reactive oxidants, complementing enzyme-mediated antioxidant defenses associated with LF and α-lactalbumin [53]. It should be noted that although camel milk contains substantially more vitamin C than bovine milk (approximately 27–34 mg/L), a 250 mL/day serving provides only about 7–9 mg. Its independent contribution to hepatic redox status therefore needs to be evaluated in relation to intake, bioavailability, and hepatic exposure; current evidence more strongly supports protein components such as LF, α-lactalbumin, and casein-derived peptides as major carriers of antioxidant activity [47,53]. These mechanisms are considered to be of great significance in the intervention process of NAFLD [48,53].

### 2.3. Osteoporosis

Osteoporosis is a metabolic bone disease characterized by decreased bone mass, deterioration of bone microstructure and increased bone fragility. Its essence is the disruption of the remodeling balance between bone formation and bone resorption [54]. The occurrence and development of osteoporosis are closely related to a variety of age-related factors. The cumulative effects of chronic inflammation, oxidative stress, and bone metabolism imbalance with age can accelerate bone loss and bone microstructure degeneration [55]. To date, no human clinical study specifically evaluating camel milk against bone mineral density, bone-turnover markers, or fracture risk was identified; the direct evidence currently comes from animal studies.

In an animal study, Sprague-Dawley rats received cow, buffalo, goat, or camel milk by gavage for six weeks. Femoral histology showed trabecular thinning and erosion with marked osteoclast infiltration in the cow-, buffalo-, and goat-milk groups, whereas the camel-milk group retained dense, intact trabeculae; normal osteocyte morphology; regular lamellae; more osteoblasts; and few osteoclasts. The authors therefore concluded that camel milk prevented histopathological changes considered early signs of osteoporosis [56]. This protective effect has a clear nutritional basis. Composition analysis showed that camel milk had the highest calcium content among the four milks, a reported vitamin D3 content 8–20 times higher, and the lowest phosphorus content [56]. Calcium and vitamin D are key substrates for bone mineralization, and their enrichment together with a lower phosphorus load creates a mineral profile favorable to bone health. Serum indices were consistent with these compositional differences. Serum 25-hydroxyvitamin D3 [25(OH)D3] was significantly higher in the camel-milk group than in controls (males 85 vs. 72 nmol/L; females 70 vs. 65 nmol/L; *p* < 0.05), whereas no increase was observed in the cow-, buffalo-, or goat-milk groups; total and ionized calcium also increased significantly in male rats (12.91 vs. 11.11 mg/dL; 1.54 vs. 1.31 mmol/L). Meanwhile, the other three milk groups significantly increased serum phosphorus (males 7.05–7.91; females 6.25–7.25 mg/dL), whereas the camel-milk group did not differ from controls (males 6.11 vs. 6.11; females 5.71 vs. 5.65 mg/dL), consistent with its lower phosphorus content. The skeletal effects of camel milk may also be related to its lipid profile. Camel milk had the lowest saturated fatty acid content (50.1%) and only 1.95% lauric acid, while showing the highest monounsaturated and polyunsaturated fatty acid contents (40.1% and 9.8%). Consistent with this, the other three milks increased total cholesterol and triglycerides, whereas camel milk did not produce the same hyperlipidemic profile and was associated with higher high-density lipoprotein cholesterol (HDL-C) and lower low-density lipoprotein cholesterol (LDL-C) and very-low-density lipoprotein cholesterol (VLDL-C) [56]. High-fat or high-cholesterol diets can downregulate genes related to transforming growth factor beta/bone morphogenetic protein (TGF-β/BMP) and Wnt signaling and suppress osteoblast proliferation and differentiation through reduced bone morphogenetic protein 2 (BMP2) expression and oxidative injury [57], while increasing bone-resorption-related gene expression and the osteoclastogenic potential of bone-marrow cells [58,59]. Because camel milk did not induce hyperlipidemia, it may avoid this lipid-mediated pathway of bone injury [56].

Camel milk is a rich source of LF, with reported concentrations of approximately 220–229 mg/L, higher than those reported for bovine milk which is approximately 140 mg/L [60,61]. In vitro studies have directly examined camel-milk-derived LF in bone cells. At 25, 50, and 100 µg/mL, camel LF significantly increased proliferation of MG-63 osteoblast-like cells relative to controls, and loading LF onto perfluorooctyl bromide nanoparticles did not abolish this proliferative activity [62]. Similar findings were reported when camel LF was encapsulated in whey-protein-isolate–pectin complexes [63]. Camel and bovine LF share 74.9% primary-sequence homology and have similar molecular weights [60]; this similarity supports cautious use of bovine LF studies as mechanistic context for camel LF rather than as direct evidence. Trybek et al. reported in a systematic review and meta-analysis that systemic or local LF administration promotes new bone formation, with stronger osteogenic effects at higher doses; the authors related this effect to a shift in the receptor activator of nuclear factor-κB (RANK)/RANK ligand (RANKL)/osteoprotegerin (OPG) system toward OPG [64]. On the osteogenic side, LF promotes proliferation and differentiation of mesenchymal stem cells and preosteoblasts through transforming growth factor beta receptor II (TβRII)-mediated Smad2 and p38 MAPK signaling; knockdown of TβRII or inhibition of transforming growth factor beta receptor I (TβRI) markedly attenuates LF-induced alkaline phosphatase (ALP) activity and expression of osteogenic markers such as osteocalcin (OCN) and osteopontin (OPN) [65]. In addition, LF has been shown to stimulate osteoblast differentiation via a low-density lipoprotein receptor-related protein 1 (LRP1)-independent mechanism, primarily through the protein kinase A (PKA)/p38 MAPK signaling cascade, which promotes Runt-related transcription factor 2 (Runx2) phosphorylation and downstream osteogenic gene expression. Notably, while LRP1 mediates LF-induced osteoblast proliferation via extracellular signal-regulated kinase 1/2 (ERK1/2) activation, it is not required for LF’s osteoinductive effect on differentiation [66]. In a model more closely resembling postmenopausal bone loss, six-month-old ovariectomized (OVX) rats given oral bovine LF for six months showed improved bone mass and microarchitecture by micro-computed tomography (micro-CT), together with an increased femoral OPG/RANKL messenger RNA (mRNA) ratio [67]. On the osteoclast side, classic in vitro studies have shown that bovine LF reduces osteoclast differentiation and bone-resorptive activity [68].

In another dose–response study in weaning rats, camel milk intake improved bone metabolism-related parameters in a dose-dependent manner, with more pronounced effects observed at higher doses [69]. The study summarized several outcomes using composite Z scores: a bone-health score based on serum total calcium, phosphorus, and calcitriol, and a vitamin D metabolism score based on calcidiol and calcitriol, each standardized to the same-sex control group. Both scores increased with dose, with large effect sizes at 3.4 mL/day and above. Because calcitriol contributed to both composite scores, their positive association partly reflected overlap in their construction; the original study did not report a correlation coefficient or independently validate the scores. The relationship therefore suggests concurrent improvement in vitamin D status and bone-related biochemical measures but is insufficient to establish mediation [69]. Numerical differences between sexes were reported for both scores, but the analysis used one-way ANOVA, and each measure was standardized to the same-sex control without a sex-by-treatment interaction test. The available data can therefore describe differences in effect size between sexes but are insufficient to establish a sex-specific biological response [69]. The authors also noted that doses above the recommended level further increased some bone-related indices but were accompanied by elevations in liver and kidney function markers; they therefore identified 3.4 mL/day as an intake that balanced efficacy and safety in this model [69].

### 2.4. Obesity and Adipose Tissue Dysfunction

Obesity is not merely an increase in body weight but rather a chronic condition characterized by excessive adipose accumulation, often accompanied by abnormal fat distribution and adipose-tissue dysfunction. The core pathology of obesity-associated metabolic disorders extends beyond simple fat accumulation to include insulin resistance, dyslipidemia (elevated TGs and reduced HDL-C), and chronic low-grade inflammation, which collectively drive systemic metabolic imbalance [12]. The accumulation of senescent cells and their senescence-associated secretory phenotype, chronic low-grade inflammation (inflammaging), mitochondrial dysfunction, impaired proteostasis, and dysregulated nutrient-sensing pathways are also recognized features of aging [12] and are amplified in obesity; obesity is therefore considered capable of accelerating aging-related phenotypes and increasing susceptibility to age-associated disease [70]. It should be emphasized that this broad mechanistic overlap does not make obesity equivalent to aging; rather, obesity can be viewed as a metabolic stress state that may accelerate aging-related phenotypes.

Human evidence for camel milk in obesity-related metabolic dysfunction is currently limited to two small trials totaling fewer than 50 participants, with intervention periods of approximately eight weeks to two months. In 24 overweight or obese adolescents with metabolic syndrome, 250 mL/day of fermented camel milk for eight weeks reduced AST by 3.75 U/L and ALT by 2.54 U/L relative to diluted cow-milk yogurt [44]; however, in companion analyses from the same trial, inflammatory cytokines (interleukin-6 (IL-6) and tumor necrosis factor alpha (TNF-α)), incretins (glucose-dependent insulinotropic polypeptide (GIP) and GLP-1), glycemic measures, body weight, body mass index (BMI), waist circumference, and other adiposity-related measures did not change significantly [45,71].

A systematic review and meta-analysis integrating cellular, animal, and human evidence reported generally favorable effects of camel-derived products on antioxidant and anti-inflammatory outcomes [15,72]. In animal models, camel-milk-related interventions have improved obesity-associated metabolic abnormalities at several levels, with direct measurements supporting effects on adiposity and energy expenditure. For example, in a rat model induced by a high-fat, high-cholesterol diet, continuous supplementation with freeze-dried fermented camel milk for 8 weeks significantly improved the lipid profile, showing a decrease in small dense low-density lipoprotein (sd-LDL) levels and triglyceride content, accompanied by a decrease in lipid peroxidation products like MDA and an enhancement in overall antioxidant capacity [73]. In high-fat-diet mice, orally administered camel milk-derived extracellular vesicles (mEVs) dose-dependently limited weight gain. Body-composition analysis showed a lower body-fat percentage and recovery of the lean-mass proportion, together with reductions in inguinal and epididymal white adipose tissue, brown adipose tissue, liver weight, and tissue triglyceride content. Histologically, adipocytes were smaller and hepatic lipid droplets were reduced, while serum glucose, triglycerides, free fatty acids, and total cholesterol also improved. Mechanistically, mEVs increased uncoupling protein 1 (UCP1) expression in brown adipose tissue and enriched thermogenic and oxidative phosphorylation (OXPHOS) gene programs. Metabolic-cage measurements further showed higher oxygen consumption and heat production in both light and dark phases without changes in food intake or spontaneous activity, and treated mice maintained core temperature better during acute cold exposure [74]. These findings indicate that the anti-obesity effect of mEVs in this model was driven mainly by enhanced brown-fat thermogenesis and whole-body energy expenditure rather than by suppression of food intake.

Based on this, researchers further evaluated the metabolic regulatory potential of different forms of camel milk products in obesity models. Animal studies suggest that specific probiotic-fermented camel milk preparations can improve obesity-associated dyslipidemia. In diet-induced obese rats, probiotic-fermented camel milk, alone or combined with intermittent fasting or Sukkari dates, improved weight gain and lipid measures [75]; in a hypercholesterolemic model, fermented camel milk containing *Bifidobacterium longum* BB536 significantly lowered total cholesterol, low-density lipoprotein cholesterol, and triglycerides while increasing high-density lipoprotein cholesterol [76], and improvements in atherogenic indices were reported with plant-sterol-enriched fermented camel milk [73]. The ATP-binding cassette G5/G8 (ABCG5/G8) transporter provides a relevant general mechanism for intestinal sterol efflux and biliary cholesterol secretion [77], but direct activation of this pathway by fermented camel milk has not yet been demonstrated. Camel-milk lipids have separately been reported to suppress the nucleotide-binding oligomerization domain-like receptor family pyrin domain containing 3 (NLRP3) inflammasome-related inflammatory responses in human macrophages [78]. These results suggest that the combined strategy of “probiotic fermentation plus camel milk matrix” may help enhance its metabolic regulatory effect.

In recent years, research on the mechanism of action of camel milk has been further extended to the level of adipose tissue function and energy metabolism. Proteomic analysis of camel-milk-derived extracellular vesicles has identified more than a thousand proteins, including recognized small-vesicle markers such as cluster of differentiation 9 (CD9), CD63, CD81, tumor susceptibility gene 101 (TSG101), a disintegrin and metalloprotease 10 (ADAM10), and heat-shock proteins HSP70/90, together with numerous metabolic enzymes [79]. Such analyses establish vesicle cargo composition rather than the functional contribution of these proteins in recipient cells; whether camel-milk extracellular vesicles participate in metabolic reprogramming by altering proteostasis or mitochondrial quality control in recipient adipocytes still requires further experimental evidence.

**Figure 1:**
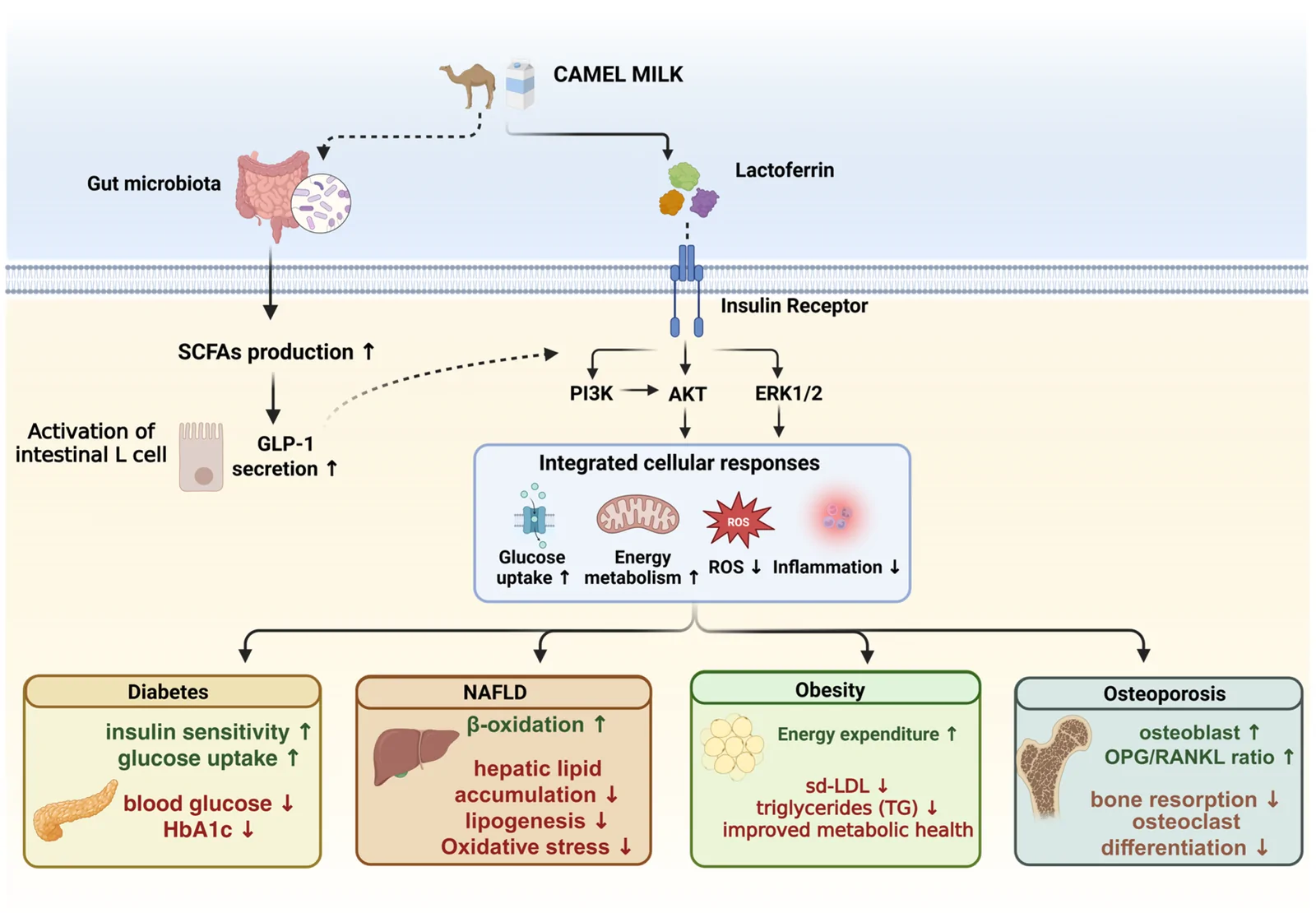
Mechanistic overview of the regulatory effects of camel milk on metabolic diseases. Note: Solid arrows indicate effects directly supported by experimental evidence from camel milk studies; dashed arrows indicate indirect or proposed links not yet directly demonstrated for camel milk. ↑ denotes an increase or upregulation and ↓ a decrease or downregulation of the indicated parameter. Created in BioRender. Cheng, M. (2026) https://BioRender.com/ywuyciq.

Overall, current evidence suggests that camel milk may serve as a potential adjunct nutritional intervention in metabolic diseases, mainly by improving key metabolic and inflammatory indicators in humans and exerting multi-target regulatory effects on glucose and lipid metabolism, oxidative stress, and tissue structure in experimental models (Figure 1).

## 3. Cardiovascular-Related Diseases

Cardiovascular disease is a group of chronic diseases characterized by abnormalities in vascular structure and cardiac function. Key pathological processes, such as endothelial dysfunction and vascular inflammatory response, are closely related to oxidative stress, activation of inflammatory signals, and lipid metabolism imbalance [12,80]. According to statistics, the prevalence and mortality of cardiovascular disease increase significantly with age and are one of the leading causes of death and disability in people over 65 years of age [80]. Current evidence suggests that camel milk may influence cardiovascular risk-related markers such as blood lipids [4]. In addition, the antioxidant peptides and a variety of micronutrients in camel milk are believed to help reduce lipid peroxidation levels, thereby alleviating the damage of oxidative stress to vascular endothelial structure and function [5].

### 3.1. Atherosclerosis

Atherosclerosis is a progressive vascular disease characterized by lipid deposition in the blood vessel walls, persistent chronic inflammation, and gradual impairment of vascular endothelial function. Its occurrence and development are often accompanied by increased oxidative stress, decreased immune regulation, and lipid metabolism imbalance, all of which collectively promote the formation of atherosclerotic plaques [81].

In population studies, clinical trials and meta-analyses in patients with diabetes have evaluated camel milk or camel milk powder against several cardiometabolic risk markers, including assessments of total cholesterol (TC) and triglycerides (TGs), and ratios such as TC/HDL-C and LDL/HDL. In a small four-week randomized controlled trial in T2DM, the camel milk powder group showed a reduction in TC from baseline, but post-intervention lipid values did not differ significantly between groups [25]; In contrast, a meta-analysis including 10 randomized controlled trials in patients with diabetes found that camel milk intake was associated with lower TC, TGs, and LDL and higher HDL, although between-study heterogeneity was high [26].

Similar trends have been further supported in animal models. In rats fed a high-fat, high-cholesterol diet, fermented camel milk reduced the atherogenic index, small dense low-density lipoprotein (sd-LDL), and serum malondialdehyde (MDA), while increasing total antioxidant capacity [73]. In male pigs with streptozotocin (STZ)- and high-fat-diet-induced metabolic injury, camel milk attenuated aortic intimal ulceration, medial cystic necrosis, and collagen-fiber disruption and reduced aortic lectin-like oxidized low-density lipoprotein receptor-1 (LOX-1) mRNA expression [82]. These findings provide evidence of effects on metabolic status, oxidative stress, aortic tissue injury, and an oxidized-LDL-related vascular marker in experimental models. From a mechanistic perspective, oxidative stress is considered to play a key role in the occurrence and progression of atherosclerosis. Oxidative modification of low-density lipoprotein can induce endothelial cell dysfunction, promote monocyte adhesion and foam cell formation, thereby accelerating plaque development [81,83]. These changes in overall levels provide experimental evidence for its potential anti-atherosclerotic effect.

Available in vitro studies indicate that camel-milk-derived lipids and low-molecular-weight peptides generated during fermentation can modulate macrophage inflammatory responses [5,78,84]. In glycated-serum-albumin-stimulated differentiated THP-1 (dTHP-1) human macrophages, camel-milk total lipids and total fatty acids reduced secretion of tumor necrosis factor alpha (TNF-α), interleukin-1 beta (IL-1β), and interleukin-18 (IL-18); total lipids also modulated nuclear factor kappa B (NF-κB), NLRP3, and caspase-1 and increased interleukin-10 (IL-10), interleukin-1 receptor antagonist (IL-1Ra), and cluster of differentiation 163 (CD163), a marker associated with an M2-like macrophage phenotype [85]. From a vascular pathology perspective, this anti-inflammatory effect helps maintain endothelial cell function, improve nitric oxide (NO) bioavailability, and may reduce the risk of plaque instability [83]. From a general vascular-mechanism perspective, oxidative stress can promote endothelial nitric oxide synthase (eNOS) uncoupling through oxidation of tetrahydrobiopterin (BH4), thereby reducing nitric oxide (NO) bioavailability [86]; in cyclosporine-treated rats, camel milk reduced renal oxidative stress and enhanced AKT/eNOS/NO signaling [87], providing indirect support for a redox-sensitive NO mechanism, although this still requires direct validation in vascular models. In addition, camel milk-derived dodecapeptide YY-11 inhibited TGF-β-induced reactive oxygen species (ROS) production and expression of genes related to glycosaminoglycan-chain elongation in human vascular smooth-muscle cells [88]; together with the macrophage and animal evidence described above, these findings provide a mechanistic basis for the possibility that camel milk may influence atherosclerosis-related processes by modulating inflammatory and oxidative-stress pathways.

### 3.2. Hypertension

Hypertension is a chronic cardiovascular disease characterized by persistently elevated arterial blood pressure, typically manifested as a long-term abnormally high systolic or diastolic blood pressure, with its prevalence increasing significantly with age. Hypertension is often accompanied by various pathological changes, including decreased vascular elasticity, endothelial dysfunction, and chronic low-grade inflammation [26].

Existing evidence regarding the relationship between camel milk and hypertension mainly comes from animal experimental models, such as spontaneously hypertensive rats (SHR). Yahya et al. reported that in 11-week-old male SHRs, single doses of 240 or 1200 mg/kg fermented skim camel milk lowered systolic and diastolic blood pressure at 4 and 8 h; with repeated administration for 20 days, both doses continued to lower systolic pressure and the 1200 mg/kg dose also significantly reduced diastolic pressure. Thus, the study supports both acute and repeated-dose antihypertensive potential of fermented skim camel milk in SHRs, with a degree of dose dependence [85]. Compared with the same high dose of unfermented skim camel milk, fermented skim camel milk produced a stronger antihypertensive effect and lowered plasma angiotensin-converting enzyme (ACE) activity in SHRs [85]. Several potential ACE-inhibitory peptides have been identified in fermented camel milk, and related casein-derived sequences show stability during simulated gastrointestinal digestion or potential for intestinal epithelial transport [47,89], supporting the possibility that fermentation-derived bioactive peptides contribute to ACE-related antihypertensive effects. In addition, in Wistar rats with hypertension and insulin resistance induced by an 8% high-salt diet, camel milk lowered systolic blood pressure and pulse rate and improved glucose, insulin, homeostatic model assessment of insulin resistance (HOMA-IR), and lipid indices [90]. Although aging was not an experimental variable in that study, the simultaneous effects on hypertension and metabolic abnormalities provide indirect preclinical evidence relevant to cardiometabolic pathways involved in vascular aging.

From a mechanistic perspective, age-related hypertension is often accompanied by increased vascular oxidative stress, chronic low-grade inflammation, and endothelial dysfunction. These factors continuously promote blood pressure elevation by reducing nitric oxide (NO) bioavailability and disrupting vasodilatory response [80]. In a study mentioned above, in high-fructose-fed adult rats, intact camel milk protein and camel milk protein hydrolysate lowered systolic and diastolic pressure together with serum ACE, angiotensin II (Ang II), and endothelin-1; changes in ROS, MDA, SOD, and reduced glutathione (GSH) were measured in liver rather than vascular tissue [50]. More direct vascular evidence comes from camel-milk-derived casein peptides: camel casein tryptic hydrolysate-induced concentration-dependent relaxation in isolated rat thoracic aorta and mesenteric artery, with the aortic effect dependent on an intact endothelium and mediated through the nitric oxide/cyclic guanosine monophosphate (NO/cGMP) pathway [91]. Because impaired nitric oxide signaling and endothelial dysfunction are core features of vascular aging [80], this vasorelaxant effect provides a mechanistic rationale for studying camel-milk-derived peptides in age-related hypertension, but it has not yet been validated in aging models or human studies [91]. At the component level, purified camel LF shows ferric-reducing and free-radical-scavenging activity and can inhibit Fe^2+^-mediated DNA damage in cell-free assays [92]. These findings support antioxidant potential for camel LF, while whole camel milk has also been shown to restore renal AKT/eNOS/NO signaling in a cyclosporine nephrotoxicity model [87]. However, this does not directly demonstrate that camel LF preserves eNOS function in hypertensive vessels.

**Figure 2:**
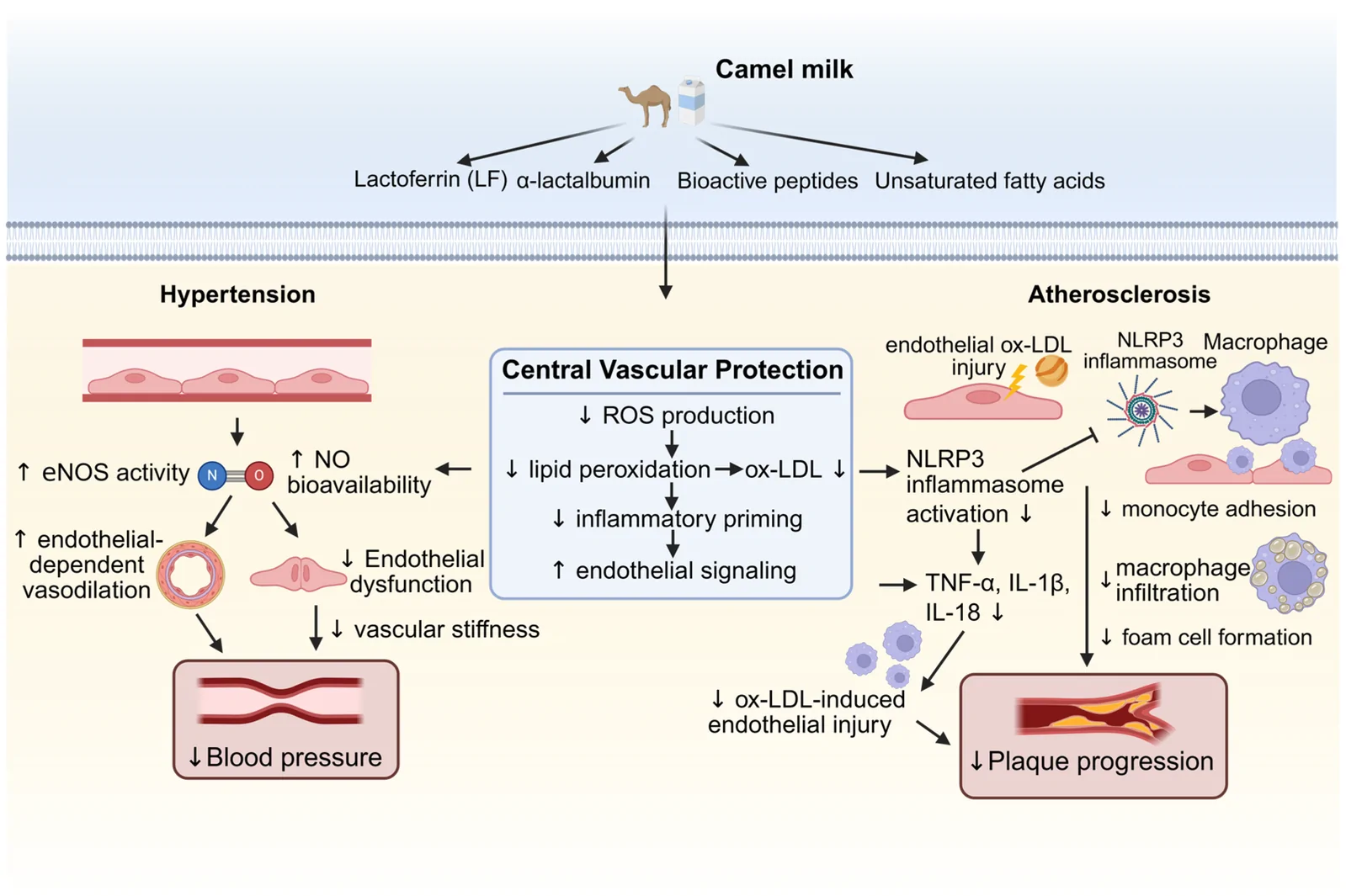
Mechanisms underlying the vascular protective effects of camel milk in cardiovascular-related diseases. Note: ↑ denotes an increase or upregulation and ↓ a decrease or downregulation of the indicated parameter. Created in BioRender. Cheng, M. (2026) https://BioRender.com/s759ow8.

All the existing studies mentioned above suggest that camel milk may play a certain auxiliary role in cardiovascular-related diseases, especially hypertension and atherosclerosis, by regulating blood lipid profiles, reducing oxidative stress and chronic inflammation, and improving vascular endothelial function (Figure 2).

## 4. Neurological Dysfunction

Neurological dysfunction is a large class of diseases or functional changes characterized by structural and functional abnormalities of the central and peripheral nervous systems. Its clinical manifestations can involve multiple dimensions such as cognition, motor function and emotional behavior [93]. Within the scope of this review, normal brain aging, age-related neurological changes, neurological dysfunction, and neurodegenerative diseases are clinically distinct concepts, but aging provides a shared biological background and is an important risk factor for neurological decline and neurodegenerative disease. With advancing age, neural plasticity and homeostatic capacity decline, while oxidative injury, chronic inflammation, and neuroimmune dysregulation accumulate; these changes not only contribute to age-related decline in cognitive, motor, and emotional functions but also increase susceptibility to neurodegenerative diseases such as Alzheimer’s disease (AD) and Parkinson’s disease (PD) [93,94,95,96,97]. Current animal and cellular studies suggest that camel milk or its derived components may influence oxidative stress, inflammation, and selected neurobehavioral and biochemical measures, which intersect with pathological processes related to neurological aging [98,99,100,101,102].

### 4.1. Age-Related Cognitive Decline

Age-related cognitive decline is a common neurological change during aging, characterized mainly by gradual reductions in memory, executive function, attention, and information-processing speed, and it can occur even in the absence of dementia [93,94]. It arises from multilevel structural and functional brain alterations, including cortical thinning, white matter integrity loss, and disrupted neural connectivity, driven by mechanisms such as immunosenescence, vascular dysfunction, and chronic neuroinflammation [95].

A preclinical study used an aluminum chloride (AlCl_3_)-induced Alzheimer-like rat model to evaluate camel milk, taurine, and their combination against neurobehavioral and biochemical outcomes; this model represents toxin-induced disease-like changes rather than physiological brain aging [98]. Compared with the AlCl_3_ model group, camel milk alone significantly prolonged motor endurance, indicating that camel milk itself improved at least some neurobehavioral end points in this model [98]. Biochemical analysis showed that amyloid beta 1-42 (Aβ1-42) concentrations were significantly reduced after camel milk alone or combined treatment; in contrast, increased superoxide dismutase (SOD) activity was observed mainly with taurine, whereas changes in acetylcholinesterase (AChE) activity were more evident with combined camel milk and taurine treatment [98]. These findings provide direct preclinical evidence that camel milk can influence Aβ-related pathology; together with antioxidant and anti-inflammatory effects reported in other animal and in vitro studies, camel milk and its bioactive components may act on pathological processes shared by brain aging and Alzheimer’s disease, including Aβ abnormalities, oxidative stress, and inflammation [5,98].

From a mechanistic perspective, age-related cognitive decline is closely associated with increased oxidative stress and chronic low-grade inflammation [5]. Based on current preclinical and in vitro evidence, the potential effects of camel milk on age-related cognitive changes may involve the combined actions of multiple constituents across redox, inflammatory, and neurochemical pathways [5,28,98,100]. First, camel milk contains LF, α-lactalbumin, β-casein, vitamin C, and other components with antioxidant activity [5,97]. In a fenpropathrin-induced neurotoxicity rat model, whole camel milk reduced brain nitric oxide and MDA levels, restored total antioxidant capacity, and attenuated neurodegenerative histopathological changes, providing direct preclinical evidence of improved redox balance in injured brain tissue [100]. Second, in the same neurotoxicity model, whole camel milk reduced brain TNF-α and increased IL-10, indicating modulation of inflammatory responses that are also involved in brain aging [100]. Third, raw camel milk and its casein fractions exhibited AChE-inhibitory activity in vitro, whereas whole camel milk partially restored brain AChE and dopamine levels in the fenpropathrin model; these findings suggest possible modulation of cholinergic and monoaminergic processes, although memory-related benefits have so far been observed only in toxin-induced models and await confirmation in natural-aging models [28,100].

### 4.2. Motor Disorders

With age, the function of the central motor control system gradually declines, especially the motor regulation network centered on the dopaminergic pathway. Degenerative changes can manifest as decreased motor coordination, bradykinesia, and weakened balance and postural control [94,103]. These age-related motor changes are not equivalent to PD, but the decline in neural plasticity, homeostatic regulation, and dopaminergic function associated with brain aging may increase vulnerability to PD-related pathology. When disease-specific processes such as α-synuclein aggregation further disrupt subcortical motor networks, reduced spontaneous activity, postural instability, and impaired motor coordination can emerge as PD-related motor phenotypes [104]. Although pharmacological and neurotoxic animal models cannot fully reproduce natural aging, they provide preclinical evidence from complementary perspectives—impaired dopaminergic regulation and stress-related brain injury—for evaluating whether camel milk can act on age-related vulnerabilities of the motor system [99,100].

Regarding dopaminergic motor regulation, an animal study using a chlorpromazine-induced Parkinson’s-like rat model evaluated the effects of camel milk on motor behavior and brain tissue pathological changes. Behavioral results showed that, compared with the chlorpromazine-only treatment group, rats in the camel milk intervention group exhibited significantly reduced catalepsy on days 21 and 30, suggesting that it may have a certain alleviating effect on PD-like motor dysfunction [99]. Brain histology also showed better overall tissue integrity and milder degenerative changes in the camel-milk group than in the chlorpromazine group [99]. In addition to the Parkinson’s-like model, Abd-Elhakim et al. further explored the neuroprotective potential of camel milk in a fenpropathrin-induced neurotoxic rat model. Notably, although this is not a direct aging model, it involves oxidative stress, inflammation, and neuronal injury, which are also important processes in brain aging. Their histopathological analysis showed that, compared with the toxin-treated group, camel milk administration could maintain the integrity of neuronal structures in brain tissue to a certain extent and reduce neurodegenerative changes, suggesting that camel milk may have a protective effect on central nervous system structures under neurotoxic injury conditions [100]. Biochemical analysis in the same study further revealed that camel milk administration restored antioxidant defenses in brain tissue, including SOD and CAT activities, while reducing MDA levels, indicating that these redox changes occurred alongside the histopathological improvements described above [100].

At the mechanistic level, research has begun to focus on the potential neuroprotective roles of specific bioactive components in camel milk. For example, camel α-lactalbumin can form a complex with oleic acid, termed the camel α-lactalbumin-oleic acid complex (CLOA); this complex has been evaluated in rotenone-treated SH-SY5Y neuroblastoma cells for its effects on oxidative stress and cellular stress defenses [101]. In this rotenone-induced injury model, CLOA showed both preventive neuroprotection and post-injury rescue effects and was accompanied by changes in sirtuin 1 (SIRT1), hypoxia-inducible factor 1 alpha (HIF-1α), forkhead box O3 (FOXO3), and heat shock factor 1 (HSF1), suggesting that SIRT1-related cellular stress networks may contribute to the ability of CLOA to reduce oxidative injury and maintain cellular homeostasis [101]. SIRT1 participates in aging-related processes including oxidative-stress regulation, inflammation, mitochondrial function, and cellular homeostasis. Thus, modulation of SIRT1-related networks by CLOA provides a mechanistic basis for the possibility that camel-milk-derived components act on vulnerabilities relevant to brain aging; however, these findings do not directly demonstrate that drinking whole camel milk activates SIRT1 in vivo [43,101]. Accordingly, [101] provides a molecular clue that a camel-milk-derived component may influence SIRT1-related aging networks. The link between whole camel milk and protection of age-related motor function, however, rests mainly on the mechanistic overlap between improvements in motor behavior, neural-tissue integrity, and antioxidant defense observed in the animal studies above and vulnerabilities of the aging brain; it still requires validation in natural-aging models and human studies [94,99,100,101].

### 4.3. Emotional Dysregulation

In animal studies, aging is associated with changes in exploratory activity, anxiety-like behavior, social novelty preference, and other emotion- or stress-related behaviors. These phenotypes reflect remodeling of behavioral regulation in the aging nervous system, but the direction of change is not uniform and may depend on the behavioral test and age-related reductions in locomotor capacity [105]. Such experimental behaviors should not be equated directly with clinical anxiety disorders and are more appropriately considered indicators of age-related changes in emotion- and stress-related regulation. Their biological context may involve age-related changes in hypothalamic–pituitary–adrenal (HPA) axis feedback and cortisol rhythms, together with oxidative injury, low-grade inflammation, and heightened neuroimmune reactivity that influence emotion and stress resilience [94,106,107].

Although there is currently a lack of research on the effects of camel milk intake on changes in human emotional state, studies have begun to assess the effects of camel milk on emotion-related behaviors in animal experiments. In a behavioral pharmacology study in adult Wistar rats, oral whole camel milk was evaluated using the elevated plus maze, open-field test, and light-dark box for anxiety-like and exploratory/locomotor behavior, while whole-brain biogenic amines were measured in a separate group. Compared with controls, after 30 days of camel milk treatment, rats showed higher open-arm time and entry-related measures in the elevated plus maze. The open-field test also showed changes in locomotor and exploratory end points, including increased total activity, peripheral activity, and rearing, although central-zone crossings did not change in the same direction [102]. Taken together, these behavioral end points indicate that whole camel milk produced neurobehavioral effects related to anxiety-like and exploratory behavior under the experimental conditions. Camel milk treatment was also accompanied by changes in whole-brain monoamine neurotransmitters and their metabolites, suggesting that the behavioral changes may be associated with altered central monoaminergic signaling [102]. Specifically, whole-brain norepinephrine increased significantly, whereas serotonin (5-hydroxytryptamine; 5-HT) did not change significantly; dopamine and its metabolites 3,4-dihydroxyphenylacetic acid (DOPAC) and homovanillic acid (HVA), together with 5-hydroxyindoleacetic acid (5-HIAA), decreased [102]. This neurotransmitter-specific pattern further suggests that camel milk may influence the central monoaminergic neurochemical environment, but it is more appropriately interpreted at present as a neurochemical feature accompanying the behavioral changes.

Meanwhile, age-associated changes in gut microbiota composition and disruptions in gut–brain axis signaling have also been implicated in alterations of emotion- and stress-related behaviors [102,106,108]. Although direct evidence is still limited, emerging data suggest that camel milk may modulate the gut microbiota and systemic inflammation, which, together with its neuropharmacological effects on biogenic amines and behavior, supports a testable hypothesis that the microbiota–gut–brain axis and emotion- or stress-related outcomes partly through modulation of the microbiota–gut–brain axis [109].

**Figure 3:**
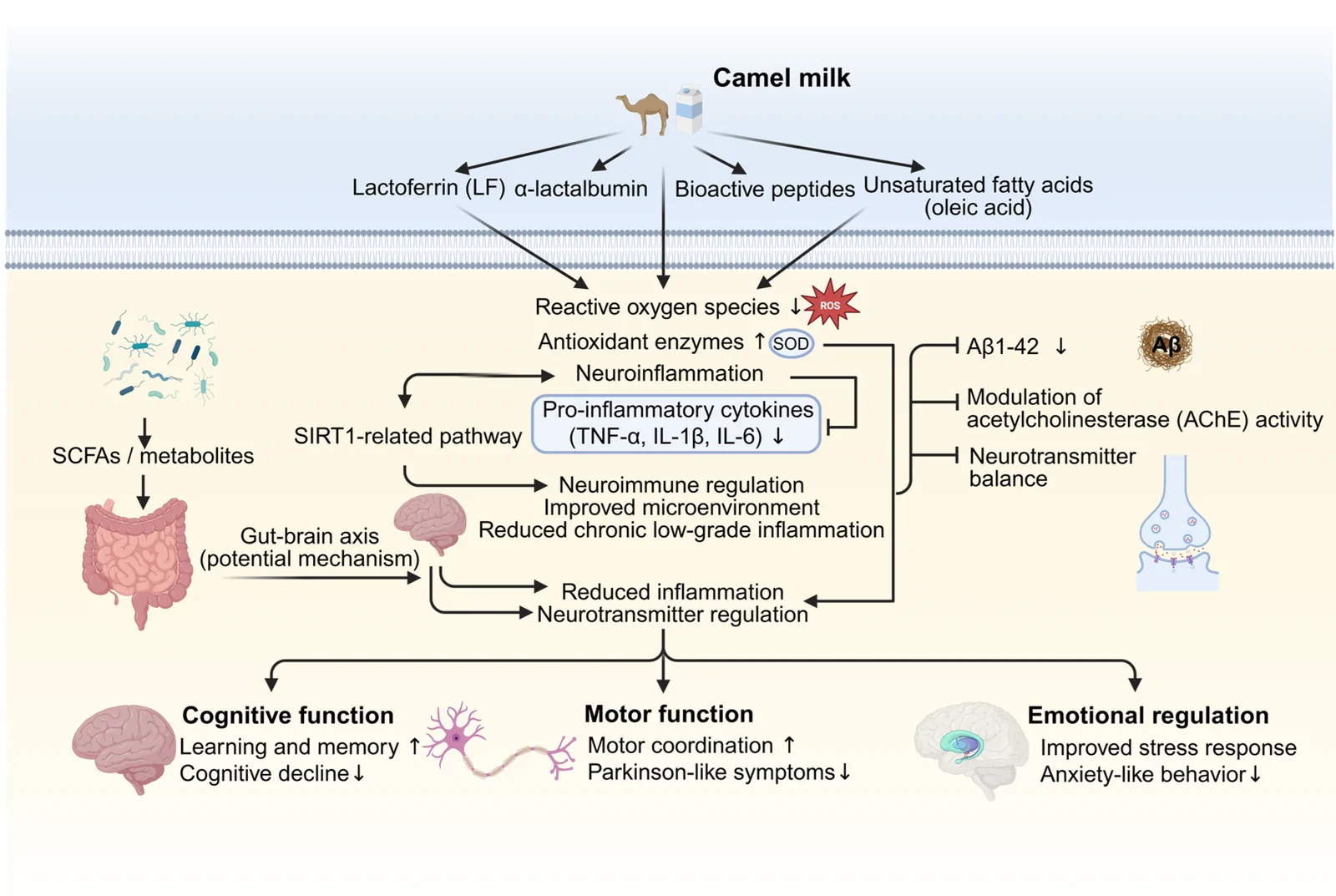
Mechanistic pathways underlying the neuroprotective effects of camel milk in neurological dysfunction. Note: ↑ denotes an increase or upregulation and ↓ a decrease or downregulation of the indicated parameter. Created in BioRender. Cheng, M. (2026) https://BioRender.com/85ejl8x.

Taken together, existing research suggests that camel milk may play a certain neuroprotective and regulatory role in neurological dysfunction, including age-related cognitive decline, motor disorders, and mood-related behavioral changes, by regulating oxidative stress, chronic inflammation, and the neuroimmune microenvironment, as well as influencing neurotransmitters and gut–brain axis-related pathways (Figure 3).

## 5. Immune-Inflammatory Disorders

Immune-inflammatory-system-related diseases are a large category of chronic diseases in which immune regulation is disrupted and inflammatory responses persist or fail to resolve [110]. These age-related immune changes can affect both innate and adaptive immunity and are accompanied by persistent production of pro-inflammatory mediators, abnormal immune-cell function, impaired resolution of inflammation, and reduced tissue-repair capacity after injury, ultimately disrupting immune-tissue homeostasis. With age, the precision of immune regulation declines, immune tolerance becomes more difficult to maintain, and inflammatory signaling may remain chronically activated at a low level, increasing susceptibility to immune-inflammatory disorders [110]. Multiple studies have shown that immune-related proteins, bioactive peptides, and antioxidant-related nutrients in camel milk have positive effects on immune homeostasis [5,111,112]. Accordingly, this section does not treat rheumatoid arthritis, allergic airway inflammation, and burns or diabetic wounds as a single clinical disease category. Instead, they are discussed as distinct disease or injury contexts in which age-related decline in immune regulation, persistence of chronic inflammation, and reduced tissue-repair capacity may be relevant.

### 5.1. Rheumatoid Arthritis

Rheumatoid arthritis (RA) is a systemic autoimmune disease characterized by chronic joint inflammation, synovial hyperplasia, and joint destruction [113]. Its development involves multi-level immune imbalances, including abnormal activation of immune cells, persistently elevated levels of pro-inflammatory cytokines, impaired anti-inflammatory regulatory pathways, and enhanced oxidative stress [111].

In adult Wistar rats (*n* = 8 per group), Arab et al. evaluated camel milk using Freund’s complete adjuvant (FCA)-induced adjuvant arthritis together with a subcutaneous air-pouch model; camel milk was administered by oral gavage at 10 mL/kg/day for 21 days, beginning 2 h before FCA inoculation [114]. Because administration began before arthritis induction, this was a preventive or early-intervention design rather than treatment of established arthritis. The results showed that continuous camel milk intervention significantly alleviated the clinical manifestations of arthritis, including improvements in limb swelling, joint function, and gait scores, accompanied by a significant reduction in inflammatory cell infiltration and histopathological damage [114]. Based on functional improvements, this study additionally analyzed changes in inflammatory and immune-related markers. Compared with the model control group, camel milk treatment significantly reduced the level of TNF-α in the peripheral inflammatory environment, while increasing the expression of the anti-inflammatory cytokine IL-10, suggesting that it can exert anti-inflammatory effects by regulating the local and systemic immune microenvironment of the joint [114]. The upregulation of IL-10 is of particular relevance in RA pathophysiology, as IL-10 has been shown to suppress synovial fibroblast-like synoviocyte (FLS) proliferation and RANKL expression, thereby limiting both the inflammatory amplification and osteoclast-mediated bone erosion that characterize progressive joint destruction [115]. Further histological and molecular analysis showed that camel milk intervention could downregulate the expression of key inflammatory proteins closely related to the progression of RA inflammation, such as NF-κB p65, cyclooxygenase-2 (COX-2), and inducible nitric oxide synthase (iNOS). These signaling molecules are considered to be important pathways driving inflammatory cell activation and tissue destruction. Western blotting of hind-paw tissue showed that camel milk reduced the phosphorylation of p38 MAPK, ERK1/2, and c-Jun *N*-terminal kinase 1/2 (JNK1/2) by 51.9%, 36.6%, and 37.4%, respectively. This coordinated regulation further suppressed downstream COX-2/prostaglandin E2 (PGE2) and iNOS/NO production, suggesting that it may attenuate joint inflammation and related oxidative stress damage through multiple inflammatory signaling axes [114].

Beyond FCA-based models, other experimental data also support a potential adjunctive role of camel milk in inflammatory rheumatic conditions [116,117]. In the collagen-induced arthritis experiment (*n* = 6 rats per group), camel milk at 5 or 10 mL/kg or the lactoferrin preparation at 100 mg/kg was administered orally from day 15 for 30 days, with oral ibuprofen at 40 mg/kg as the reference treatment. Relative to the disease-control group, camel milk and lactoferrin were associated with lower arthritis indices and paw thickness and with improved radiographic joint scores [117]. Taken together, these pleiotropic actions align closely with key pathogenic pathways in RA, providing a biological rationale for considering camel milk as a potential adjunctive strategy in the inflammatory control of RA.

### 5.2. Allergic Diseases

The occurrence of allergic inflammation is closely related to impaired immune tolerance, a bias towards T helper 2 (Th2)-type immune responses, and abnormalities in immunoglobulin E (IgE) synthesis and regulation. Aging, however, does not uniformly amplify allergic immunity. Immunosenescence and inflammaging remodel innate and adaptive immune responses as well as the airway and epithelial microenvironment, while several measures of allergen sensitization and IgE-associated reactivity decline with advancing age [118,119,120]. Accordingly, allergen sensitization is generally less prevalent at advanced ages, but clinically relevant atopy persists; among older adults with asthma, airway inflammation may shift toward mixed eosinophilic–neutrophilic patterns associated with poorer disease control [119,120]. Allergic disease in later life should therefore be regarded as a heterogeneous phenotype shaped by persistent or late-onset sensitization, age-related airway and epithelial-barrier changes, chronic inflammation, cumulative environmental exposures, comorbidities, and medication use [119,120].

A recent study evaluated the preventive effects of camel milk on house dust mite (HDM)-induced allergic airway inflammation in 8–10-week-old female BALB/c mice. Camel milk (0.5 mL) was administered by oral gavage five times per week, beginning one day before HDM sensitization. Oral camel milk intervention significantly reduced airway inflammation, manifested as decreased eosinophil infiltration in lung tissue and suppression of Th2-type immune responses, which was further supported by reductions in pulmonary Th2 and T helper 17 (Th17) cell populations, decreased IL-4 and IL-13 expression upon HDM restimulation in vitro, lower overall levels of IL-5 and IL-13 [121]. More importantly, the simultaneous reduction in pulmonary Th2 and T helper 17 (Th17) cells may be mechanistically relevant to aging-related allergic airway disease because aging can remodel allergen-induced inflammation from a predominantly Th2 response toward a mixed Th2/Th17 phenotype. In HDM models comparing different ages, older mice show stronger Th17-related inflammation, greater airway hyperresponsiveness, and mixed eosinophilic-neutrophilic inflammation; bronchial epithelial responses in older animals also show a relatively stronger Th17 than Th2 tendency after HDM-related stimulation [122]. Thus, the ability of camel milk to attenuate both Th2- and Th17-associated responses suggests that it may act on immune pathways involved in age-related remodeling and persistence of allergic airway inflammation. In addition, prophylactic oral administration of camel whey protein can reduce serum IgE levels in mouse IgE-dependent and IgE-independent allergic reaction models and inhibit mast cell degranulation [123]. This possibility still requires direct testing in aged animal models, but the available evidence provides a mechanistic basis for considering camel milk in the context of aging-related allergic disease.

From the perspective of disease mechanism, the occurrence and maintenance of allergic diseases are not driven by a single abnormal immune response, but are closely related to the disruption of immune homeostasis, the continuous amplification of inflammatory response, and the impaired establishment or maintenance of immune tolerance. Akdis et al.’s review pointed out that chronic inflammatory background and decreased immune regulation capacity are important basic factors that make it difficult to terminate allergic reactions [124]. In this context, the inflammation relief and immunomodulatory effects mediated by camel milk observed in the aforementioned animal studies are consistent with the key pathological features of allergic diseases at the mechanistic level; that is, by improving the overall immune regulatory environment, rather than simply inhibiting the immune response, it may help to relieve allergic inflammation.

### 5.3. Barrier Dysfunction

Normal wound healing depends on the timely resolution of inflammation, followed by cell proliferation, angiogenesis, re-epithelialization, extracellular-matrix remodeling, and other coordinated processes. Aging slows these repair dynamics and is accompanied by reduced fibroblast and keratinocyte proliferation, delayed re-epithelialization, impaired collagen formation, and poorer vascular repair [125]. Persistent low-grade inflammation, cellular senescence, and redox imbalance further reduce tissue-repair resilience in older tissues, making delayed resolution, regeneration, and remodeling more likely after injury [126,127].

At the animal experimental level, studies have evaluated whole camel milk as well as extracellular vesicles and peptide preparations isolated from camel milk against barrier-repair processes in different tissue-injury models. Azaryan et al. isolated camel-milk exosomes (CM-Exo) and evaluated their repair effects in human dermal fibroblasts in vitro and in a rat burn-wound model; the animal experiment used a topical preparation combining a high concentration of CM-Exo with 1% silver sulfadiazine cream [128]. This preparation accelerated reduction of the burn-wound area and improved histological healing; in vitro, CM-Exo increased human dermal fibroblast viability and scratch closure and affected interleukin-6 (IL-6) and vascular endothelial growth factor A (VEGF-A) expression. This effect is further associated with activation of the AKT/HIF-1α signaling pathway, which promotes angiogenesis and fibroblast proliferation, along with downregulation of IL-6 and TNF-α and modulation of the TGF-β3/Smad3 axis, ultimately contributing to improved histological healing [129]. Furthermore, this study also showed that CM-Exo can alleviate oxidative stress damage, reduce ROS-related indicators, and promote skin tissue repair and regeneration [128,129].

In addition to extracellular vesicles, Ebaid et al. studied camel milk peptides (CMP) generated by proteolysis in a full-thickness skin-wound model using 8-week-old diabetic Sprague-Dawley rats; oral gavage of CMPs at 25 mg/kg/day for seven consecutive days markedly accelerated wound closure [130]. Based on this, histological analysis showed that it promoted collagen deposition and tissue remodeling, thereby improving overall wound healing efficiency against the backdrop of metabolic abnormalities [130]. In another STZ-induced diabetic mouse wound model, oral undenatured camel-milk-derived whey protein significantly modulated the local inflammatory response, reduced persistently elevated TNF-α, IL-1β, and IL-6, restored the reduced anti-inflammatory cytokine IL-10, and accelerated wound closure [131]. In a separate dextran sulfate sodium (DSS) colitis study, researchers compared oral whole camel milk, intravenously administered camel-milk extracellular vesicles, and an intravenously administered synthetic microRNA-148a-3p (miR-148a-3p) agomir. All three interventions improved selected colitis and barrier outcomes, while extracellular-vesicle and miR-148a-3p treatments were associated with restoration of zonula occludens-1 (ZO-1), changes in SIRT1/NF-κB signaling, and altered macrophage phenotypes [52]. Taken together, these preclinical studies suggest that whole camel milk and isolated camel-milk peptides or extracellular-vesicle preparations can influence inflammation, redox homeostasis, cell proliferation, and restoration of barrier structure, but the routes of administration and exposure differ substantially among preparations. In the context of aging, these findings are best regarded as mechanistic evidence that camel-milk-related interventions may affect age-sensitive tissue-repair pathways; topically or intravenously delivered isolated components should not be treated as equivalent to ordinary dietary camel milk intake.

**Figure 4:**
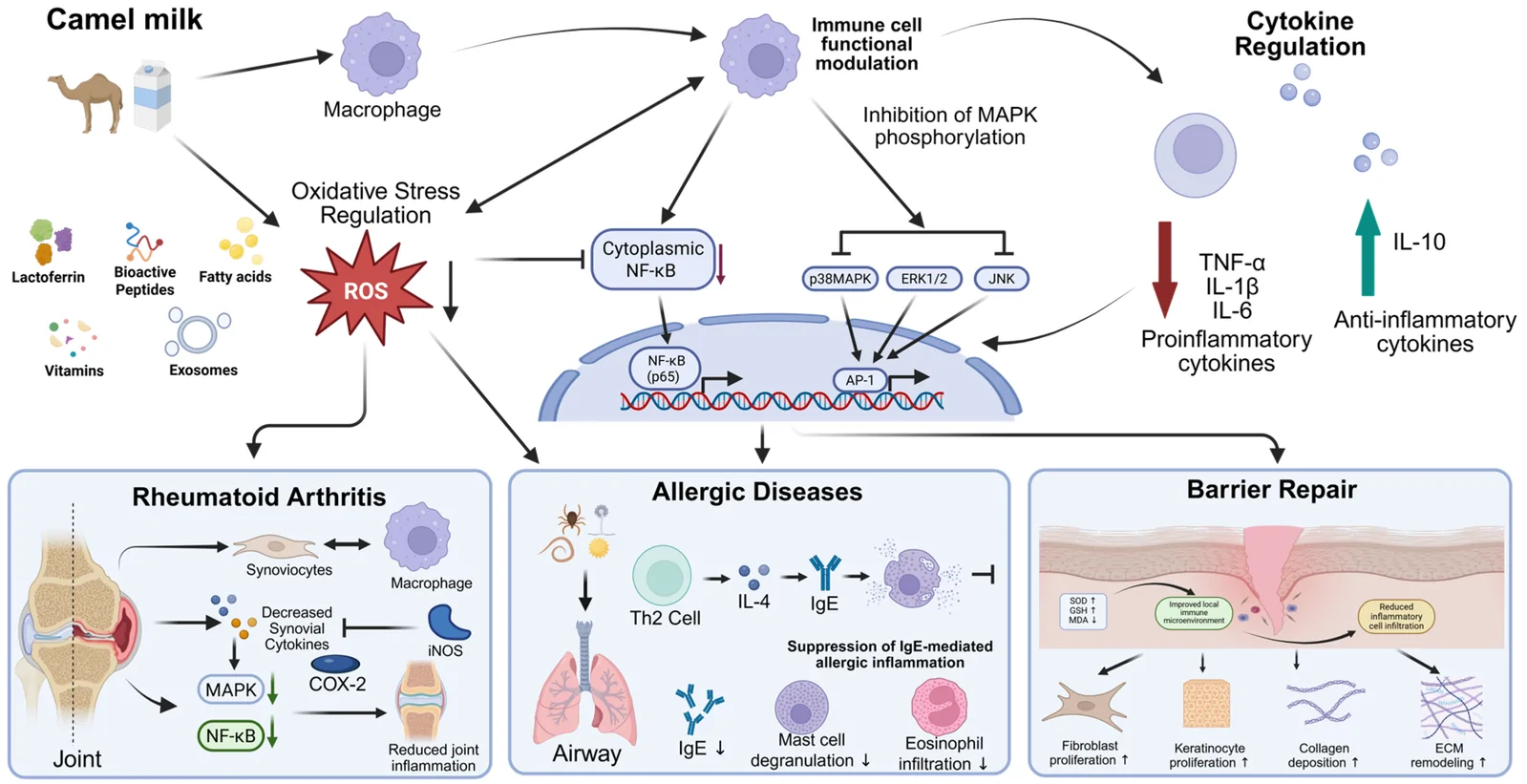
Mechanisms underlying the immunomodulatory and anti-inflammatory effects of camel milk in immune-related disorders. Note: ↑ denotes an increase or upregulation and ↓ a decrease or downregulation of the indicated parameter. Created in BioRender. Cheng, M. (2026) https://BioRender.com/ew4t57l.

Existing research suggests that camel milk may play a certain auxiliary role in immune-inflammatory diseases such as rheumatoid arthritis, allergic diseases, and skin-intestinal barrier damage by regulating immune cell function, balancing pro-inflammatory and anti-inflammatory factors, inhibiting key inflammatory pathways, and reducing oxidative stress (Figure 4).

## 6. Discussion

The main value of this review is to compare whole camel milk and its isolated bioactive components within a unified evidence hierarchy and to use the twelve hallmarks of aging as a conceptual framework to organize and interpret existing research. Table 1 provides a structured summary of the existing evidence. Among the current accumulated research evidence, the intervention studies of camel milk for diabetes stand out in terms of the availability of evidence across multiple levels and currently represent one of the areas closest to clinical translation. Existing evidence includes findings from in vitro mechanistic studies, animal models, and human clinical studies, but these levels do not yet constitute a continuous translational evidence chain. Multiple randomized controlled trials and population intervention studies have reported improvements in fasting blood glucose, postprandial blood glucose, and HbA1c levels after camel milk intervention, and some studies have also observed a reduction in the dosage of exogenous insulin or oral hypoglycemic drugs [22,23,24,25,26,132]. At the mechanistic level, multiple pathways, such as LF-mediated activation of the IR/PI3K/AKT signaling pathway, gut microbiota remodeling and regulation of short-chain fatty acid metabolism, and the synergistic relief of insulin resistance by antioxidant and anti-inflammatory effects, are suggested by experimental data, providing a biologically plausible but not yet causally validated explanatory framework [28,29,31]. However, the interventions used across these evidence levels are not equivalent: human studies generally examine whole camel milk, whereas many mechanistic studies use isolated lactoferrin, protein hydrolysates, peptides, or extracellular vesicles, with different doses, routes of administration, and exposure levels.

Human evidence, except in diabetes, is even more limited. Studies of metabolic dysfunction-associated steatotic liver disease and obesity-related metabolic dysfunction have mainly involved a small number of short-term, small-sample trials [44,45,71]. Cardiovascular studies have primarily evaluated blood lipids, blood pressure, and other risk factors rather than clinical events. Evidence related to osteoporosis, neurological dysfunction, rheumatoid arthritis, allergic diseases, and barrier dysfunction is mainly derived from animal or cellular models. Therefore, the available evidence is insufficient to support definite preventive or therapeutic effects of camel milk in these diseases.

Based on the discussions in each section, the biological effects of camel milk observed in different disease models show recurring changes in several overlapping biomarkers and signaling pathways, although this does not establish mechanistic convergence. Changes in oxidative stress-related markers are among the most frequently reported findings to date. In models of metabolic diseases, cardiovascular diseases, neurological dysfunction, and barrier repair, camel milk intervention has shown a trend of reducing lipid peroxidation products and increasing the activity of antioxidant enzymes such as SOD and GSH, indicating convergence at the biomarker level rather than establishing the ROS/antioxidant defense axis as a common causal mechanism. Changes in chronic inflammatory signaling represent another recurring pattern. Downregulation of pro-inflammatory factors such as TNF-α, IL-1β, and IL-6 has been reported in RA, atherosclerosis, allergic inflammation, and barrier damage models, accompanied by upregulation of the anti-inflammatory factor IL-10. At the signaling pathway level, the NF-κB and MAPK pathways (p38, ERK1/2, JNK1/2) have been observed to be regulated by camel milk in both RA and cardiovascular models, but these observations do not establish that these pathways mediate the anti-inflammatory effects of camel milk. Metabolic homeostasis regulation pathways—represented by PI3K/AKT, PPAR-α/γ, and AMPK-related signals—are concentrated in metabolic disease models and extend to the cardiovascular and nervous systems [31]. Activation of the PI3K/AKT pathway has been observed in insulin signaling studies; activation of the PPAR-α/γ pathway has been reported in NAFLD models; and the SIRT1-related pathway has been mentioned in component studies of neurodegenerative diseases. The gut microbiota–host metabolic axis, as an emerging mechanism, has been implicated in diabetes, NAFLD, and intestinal barrier repair models [41,89]. These observations suggest possible interactions among oxidative stress, inflammatory, metabolic, and immune-related processes, but the direction and causal relationships among these pathways remain unresolved.

**Figure 5:**
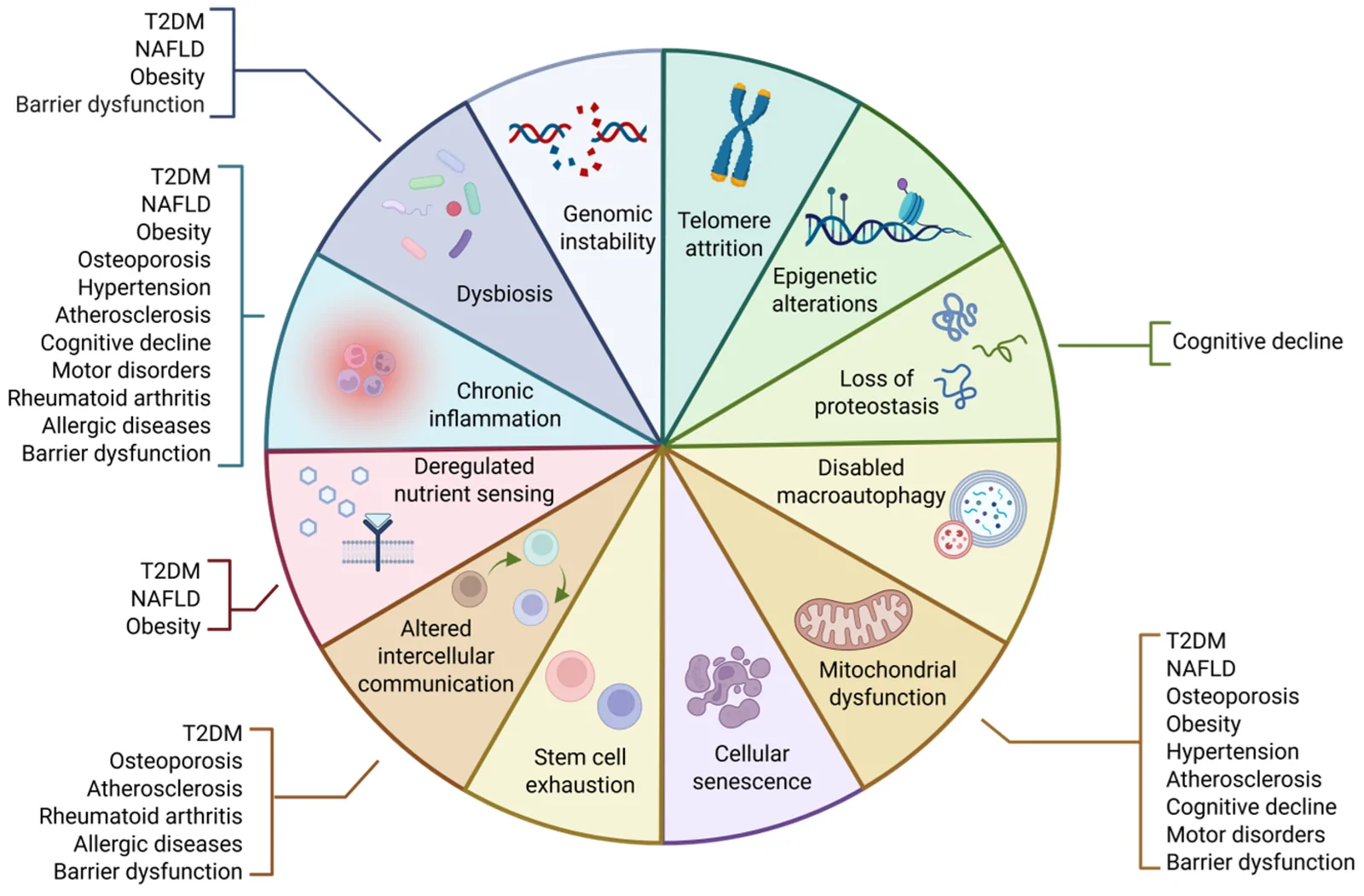
Mapping of camel milk interventions to the 12 hallmarks of aging and associated age-related diseases. Created in BioRender. Cheng, M. (2026) https://BioRender.com/7zwgb12.

In 2013, López-Otín et al. proposed nine hallmarks of aging, which were later updated to twelve [12,43]. The age-related diseases involving camel milk in this review can be considered within the aging mechanism framework proposed therein. Based on the discussion in each section of this review, we have made a preliminary summary of the conceptual links between camel milk intervention and the twelve hallmarks of aging, as shown in Figure 5. The most frequently investigated areas include chronic inflammation, oxidative stress-related changes, metabolic homeostasis dysregulation, and immune dysregulation; however, the available evidence mainly reflects changes in related biomarkers in disease models rather than direct modification of the hallmarks of aging. In addition, the nutritional and bioactive composition of camel milk may be influenced by breed, feeding conditions, geographical origin, lactation stage, and storage conditions. Pasteurization, heating, drying, and fermentation may also alter protein structure, peptide profiles, and extracellular-vesicle integrity [2,3,28]. For large-scale population use, standardized production must also be considered. However, there is currently insufficient evidence to determine which product form can preserve biological activity while meeting the requirements for safe and reproducible long-term use.

Based on the discussion above, future research should first establish a standardized framework for camel milk product characterization and quality control to improve research reproducibility. Second, preregistered, adequately powered, and appropriately controlled randomized clinical trials are needed. In addition to short-term biochemical indicators, clinically meaningful outcomes, quality of life, and physical function should also be assessed. Furthermore, mechanistic studies need to establish a continuous validation pathway. Simulated digestion, pharmacokinetic, and tissue-distribution studies should determine whether candidate components reach target tissues in biologically active forms.

## 7. Conclusions

At the human evidence level, the most substantial, although still limited, evidence comes from diabetes studies. Small trials have reported reductions in HbA1c and insulin requirements in type 1 diabetes and improvements in selected glycemic and lipid parameters in type 2 diabetes when camel milk was used as an adjunct to conventional treatment. However, substantial heterogeneity among studies precludes firm conclusions regarding consistent clinical efficacy. For obesity, metabolic dysfunction-associated steatotic liver disease, cardiovascular disease, and other age-associated conditions, human evidence remains limited, and most studies have not evaluated disease progression or clinical events.

At the animal and cellular levels, whole camel milk and isolated camel-milk components, including lactoferrin, whey protein, peptides, and extracellular vesicles, have shown biological effects in models of metabolic disorders, osteoporosis, atherosclerosis, hypertension, neurological dysfunction, immune-inflammatory conditions, and barrier injury. However, these findings are strongly dependent on the model, product, dose, and route of administration. In particular, the effects of isolated components administered intravenously or locally cannot be directly equated with those of orally consumed whole camel milk.

At the mechanistic level, changes in oxidative stress, inflammatory signaling, nutrient sensing, and the gut microbiota provide biological plausibility for the potential effects of camel milk on age-associated diseases. However, most of this evidence remains associative. Several proposed mechanisms, including effects on endothelial nitric oxide synthase coupling, in vivo activation of SIRT1-related signaling, and the functional cross-species transfer of dietary miR-148a-3p, remain unverified in the context of orally consumed whole camel milk. The proposed links between camel milk and the twelve hallmarks of aging therefore remain conceptual and hypothesis-generating rather than directly demonstrated experimentally.

Overall, camel milk may be considered a candidate dietary intervention and a source of bioactive components worthy of further investigation, but it should not currently be presented as a proven treatment for disease or as an anti-aging strategy. Standardized product characterization, rigorously designed long-term human trials, and causal mechanistic studies linking in vivo exposure, target engagement, and clinical outcomes will be essential for determining its clinical relevance and potential contribution to healthy aging.

## Figures and Tables

**Table 1:** Evidence hierarchy for camel milk and camel-milk-derived components across aging-related disease contexts.

Disease Context	Human Studies	Animal Studies	In Vitro Studies
Diabetes mellitus	Multiple intervention studies and randomized controlled trials in patients with T1DM or T2DM have evaluated glycemic indices, lipid profiles, and insulin requirements [22,23,24,25,26,132].	Diabetic and diabetes complication models have shown improvements in glycemia, insulin sensitivity, and oxidative and inflammatory indices [30,31,37,39].	Camel lactoferrin activated IR/PI3K/AKT and ERK1/2 signaling, while camel-milk-derived hydrolysates inhibited DPP-4 activity [27,28,35,36].
NAFLD/MASLD	A small crossover trial in overweight or metabolic syndrome reported improvements in liver enzymes [44].	High-fat- and high-cholesterol-diet models have shown reduced hepatic steatosis, liver injury, oxidative stress, and inflammatory changes [48,49,50,51].	No relevant in vitro model was identified.
Obesity and adipose-tissue dysfunction	Small, short-term trials involving fewer than 50 participants have been conducted in overweight, obese, or metabolic syndrome populations, with limited and inconsistent effects on adiposity related outcomes [44,45,71].	Fermented camel milk and camel-milk-derived extracellular vesicles have improved lipid profiles, adiposity, thermogenesis, and energy expenditure in diet-induced obesity models [73,74,75,76].	Mechanistic evidence remains limited; available macrophage and vesicle-cargo studies [78,79].
Osteoporosis	No human intervention study was identified.	Camel milk improved bone histology and selected biochemical indices of bone metabolism in rat models [56,69].	Camel lactoferrin promoted the proliferation of MG-63 osteoblast-like cells [62,63].
Atherosclerosis	Human studies in patients with diabetes have reported changes in lipid related cardiovascular risk markers, but have not evaluated plaque burden, vascular injury, or cardiovascular events [25,26].	Animal studies have reported improvements in atherogenic indices, aortic injury, oxidative stress, and LOX-1 expression [73,82].	Camel-milk-derived lipids, fatty acids, and peptides have modulated inflammatory and oxidative responses in macrophages and vascular smooth muscle cells [78,88].
Hypertension	No human intervention study was identified.	Fermented camel milk and camel-milk-derived preparations reduced blood pressure and ACE-related indices in hypertensive animal models [50,85,90].	Camel casein-derived peptides and hydrolysates showed ACE-inhibitory activity or NO/cGMP-dependent vasorelaxation in vitro or ex vivo [89,91].
Cognitive decline	No human intervention study was identified.	Camel milk affected Aβ1-42 concentrations and selected neurobehavioral, oxidative, inflammatory, and histopathological outcomes in toxin-induced rat models [98,100].	Raw camel milk and casein fractions exhibited AChE-inhibitory activity in vitro [28].
Motor disorders	No human intervention study was identified.	Camel milk reduced chlorpromazine-induced catalepsy and attenuated brain histopathological injury in rat models [99,100].	The camel α-lactalbumin–oleic acid complex protected rotenone-treated SH-SY5Y cells [101].
Emotional dysregulation	No human intervention study was identified.	Camel milk altered anxiety-like and exploratory behaviors and brain monoamine profiles in adult rats [102].	No relevant in vitro model was identified.
Rheumatoid arthritis	No human intervention study was identified.	Camel milk improved arthritis scores, joint pathology, inflammatory cell infiltration, and inflammatory signaling in FCA and collagen-induced arthritis models [114,117].	No relevant in vitro model was identified.
Allergic diseases	No human intervention study was identified.	Camel milk reduced Th2/Th17 associated airway inflammation in an HDM model [121]; camel whey protein reduced serum IgE and inhibited mast cell degranulation in systemic anaphylaxis models [123].	No relevant in vitro model was identified.
Barrier dysfunction	No human intervention study was identified.	Whole camel milk, peptides, whey protein, and extracellular vesicles improved wound healing or intestinal barrier outcomes in burn, diabetic wound models [52,127,129,131,133].	Camel-milk-derived extracellular vesicles increased human dermal fibroblast viability and scratch closure in vitro [128].

## Data Availability

No new data were created or analyzed in this study. Data sharing is not applicable to this article.

## Abbreviations

The following abbreviations are used in this manuscript and figures:

ABCG5/G8	ATP-binding cassette subfamily G member 5/8
ACC	Acetyl-CoA carboxylase
ACE	Angiotensin-converting enzyme
AChE	Acetylcholinesterase
AD	Alzheimer’s disease
ADAM10	A disintegrin and metalloprotease 10
AlCl_3_	Aluminum chloride
ALP	Alkaline phosphatase
ALT	Alanine aminotransferase
AMPK	AMP-activated protein kinase
Ang II	Angiotensin II
AP-1	Activator protein 1
AST	Aspartate aminotransferase
Aβ/Aβ1-42	Amyloid beta/amyloid beta 1-42
BH4	Tetrahydrobiopterin
BMI	Body mass index
BMP2	Bone morphogenetic protein 2
CAT	Catalase
CD	Cluster of differentiation
cGMP	Cyclic guanosine monophosphate
CLOA	Camel α-lactalbumin-oleic acid complex
CM-Exo	Camel milk exosomes
CMH	Camel milk hydrolysate
CMP	Camel milk peptide
COX-2	Cyclooxygenase-2
CPT1	Carnitine palmitoyltransferase-1
DOPAC	3,4-Dihydroxyphenylacetic acid
DPP-4	Dipeptidyl peptidase-4
DSS	Dextran sulfate sodium
ECM	Extracellular matrix
eNOS	Endothelial nitric oxide synthase
ERK1/2	Extracellular signal-regulated kinase 1/2
ET-1	Endothelin-1
FCA	Freund’s complete adjuvant
FLS	Fibroblast-like synoviocyte
FOXO3	Forkhead box O3
G6Pase	Glucose-6-phosphatase
GIP	Glucose-dependent insulinotropic polypeptide
GLP-1	Glucagon-like peptide-1
GLUT4	Glucose transporter type 4
GPR41/43	G protein-coupled receptor 41/43
GPx	Glutathione peroxidase
Grb2	Growth factor receptor-bound protein 2
GSH	Reduced glutathione
HbA1c	Glycated hemoglobin
HDL-C	High-density lipoprotein cholesterol
HDM	House dust mite
HIF-1α	Hypoxia-inducible factor 1 alpha
HOMA-IR	Homeostatic model assessment of insulin resistance
HPA	Hypothalamic–pituitary–adrenal
HSF1	Heat shock factor 1
HVA	Homovanillic acid
IgE	Immunoglobulin E
IL	Interleukin
iNOS	Inducible nitric oxide synthase
IR	Insulin receptor
IRS-1	Insulin receptor substrate-1
JNK/JNK1/2	c-Jun *N*-terminal kinase 1/2
LDL	Low-density lipoprotein
LDL-C	Low-density lipoprotein cholesterol
LF	Lactoferrin
LOX-1	Lectin-like oxidized LDL receptor-1
LRP1	Low-density lipoprotein receptor-related protein 1
MASLD	Metabolic dysfunction-associated steatotic liver disease
MDA	Malondialdehyde
mEVs	Milk-derived extracellular vesicles
miR-148a-3p	MicroRNA-148a-3p
NAFLD	Non-alcoholic fatty liver disease
NE	Norepinephrine
NF-κB/NF-κB p65	Nuclear factor kappa B/its p65 subunit
NLRP3	NOD-like receptor family pyrin domain containing 3
NO	Nitric oxide
OCN	Osteocalcin
OPG	Osteoprotegerin
OPN	Osteopontin
OVX	Ovariectomized
ox-LDL	Oxidized low-density lipoprotein
OXPHOS	Oxidative phosphorylation
PD	Parkinson’s disease
PEPCK	Phosphoenolpyruvate carboxykinase
PGE2	Prostaglandin E2
PKA	Protein kinase A
RA	Rheumatoid arthritis
RANK	Receptor activator of nuclear factor-κB
RANKL	Receptor activator of nuclear factor-κB ligand
ROS	Reactive oxygen species
Runx2	Runt-related transcription factor 2
RXR	Retinoid X receptor
SCFAs	Short-chain fatty acids
sd-LDL	Small dense low-density lipoprotein
SHR	Spontaneously hypertensive rat
SIRT1	Sirtuin 1
SOD	Superoxide dismutase
SREBP-1c	Sterol regulatory element-binding protein 1c
STZ	Streptozotocin
T1DM	Type 1 diabetes mellitus
T2DM	Type 2 diabetes mellitus
TC	Total cholesterol
TGs	Triglycerides
TGF-β	Transforming growth factor beta
Th2/Th17	T helper type 2/17 cells
TLR4	Toll-like receptor 4
TNF-α	Tumor necrosis factor alpha
TSG101	Tumor susceptibility gene 101
TβRI/TβRII	Transforming growth factor beta receptor I/II
UCP1	Uncoupling protein 1
VEGF-A	Vascular endothelial growth factor A
VLDL-C	Very-low-density lipoprotein cholesterol
ZO-1	Zonula occludens-1
